# Analysis of candidate genes for macular telangiectasia type 2

**Published:** 2010-12-14

**Authors:** Nancy L. Parmalee, Carl Schubert, Joanna E. Merriam, Kaija Allikmets, Alan C. Bird, Mark C. Gillies, Tunde Peto, Maria Figueroa, Martin Friedlander, Marcus Fruttiger, John Greenwood, Stephen E. Moss, Lois E.H. Smith, Carmel Toomes, Chris F. Inglehearn, Rando Allikmets

**Affiliations:** 1 Department of Ophthalmology, Columbia University, New York, NY; 2 Department of Genetics and Development, Columbia University, New York, NY; 3 Moorfields Eye Hospital, London, UK; 4 Save Sight Institute, Department of Clinical Ophthalmology and Eye Health, The University of Sydney, Sydney, Australia; 5 The EMMES Corporation, Rockville, MD; 6 Department of Cell Biology, The Scripps Research Institute, La Jolla, CA; 7 Department of Cell Biology, University College London Institute of Ophthalmology, London, UK; 8 Department of Ophthalmology, Harvard Medical School, Children's Hospital Boston, Boston, MA; 9 Section of Ophthalmology and Neuroscience, Leeds Institute of Molecular Medicine, St James's University Hospital, Leeds, UK; 10 Department of Pathology and Cell Biology, Columbia University, New York, NY

## Abstract

**Purpose:**

To find the gene(s) responsible for macular telangiectasia type 2 (MacTel) by a candidate-gene screening approach.

**Methods:**

Candidate genes were selected based on the following criteria: those known to cause or be associated with diseases with phenotypes similar to MacTel, genes with known function in the retinal vasculature or macular pigment transport, genes that emerged from expression microarray data from mouse models designed to mimic MacTel phenotype characteristics, and genes expressed in the retina that are also related to diabetes or hypertension, which have increased prevalence in MacTel patients. Probands from eight families with at least two affected individuals were screened by direct sequencing of 27 candidate genes. Identified nonsynonymous variants were analyzed to determine whether they co-segregate with the disease in families. Allele frequencies were determined by TaqMan analysis of the large MacTel and control cohorts.

**Results:**

We identified 23 nonsynonymous variants in 27 candidate genes in at least one proband. Of these, eight were known single nucleotide polymorphisms (SNPs) with allele frequencies of >0.05; these variants were excluded from further analyses. Three previously unidentified missense variants, three missense variants with reported disease association, and five rare variants were analyzed for segregation and/or allele frequencies. No variant fulfilled the criteria of being causal for MacTel. A missense mutation, p.Pro33Ser in frizzled homolog (*Drosophila*) 4 (*FZD4*), previously suggested as a disease-causing variant in familial exudative vitreoretinopathy, was determined to be a rare benign polymorphism.

**Conclusions:**

We have ruled out the exons and flanking intronic regions in 27 candidate genes as harboring causal mutations for MacTel.

## Introduction

Macular telangiectasia type 2 (MacTel) is a rare, adult onset retinal disease that results in tortuous and dilated retinal vessels, macular pigment changes, macular edema, and in some cases macular holes. It is also referred to in the literature as idiopathic juxtafoveal, or juxtafoveolar, telangiectasia. A classification system was introduced by Gass and Blodi [1] and updated in 2006 by Yannuzzi [2], distinguishing the features of three types of macular telangiectasias. MacTel, or type 2, is a bilateral disease that affects both genders, as opposed to type 1, which is often unilateral, with aneurism and exudates, and generally presents only in men. Type 3, characterized by occlusive telangiectasia, is very rare. The three forms of idiopathic macular telangiectasia are described together to distinguish and classify phenotypically similar pathologies; however, the findings and progression of the three are distinct, and it is believed that each arises from a distinct etiology.

In 2005, The MacTel Project—an international consortium of clinicians and basic science researchers—was established to study the cause, natural history, progression, and epidemiology of the disease, and to explore potential treatments. Publications from collaborators in the MacTel Project have further described the clinical findings, diagnostic methods, and epidemiology related to the disease [3-11]. In advanced cases, neovascularization may be present, arising from the intraretinal vascular plexus. While most reported cases are sporadic, affected sib pairs and affected pairs of monozygotic twins were described in the literature [12,13], leading to the hypothesis that MacTel had a genetic cause in a significant proportion of cases. Ophthalmic examination of relatives of MacTel patients revealed that many families had family members who experienced no vision loss, but did exhibit subtle signs of the disease [14]. Since vertical transmission is observed in several families, an autosomal dominant model of Mendelian inheritance with reduced penetrance is assumed.

In conjunction with the MacTel Project, we have assembled a cohort of MacTel patients and their relatives for a genetic study. One mode of investigation in our studies has been to identify the disease-causing gene(s) by the direct sequencing of candidate genes in family probands, followed by segregation analysis of families. The current hypothesis is that a dominant causal mutation would be a rare heterozygous variant in all affected individuals. Allele frequencies were determined by screening cases and controls for unknown variants, and for known variants where population frequency data was unavailable.

We selected candidate genes that were known to be causative in diseases with phenotypic similarity to MacTel (e.g., familial exudative vitreoretinopathy [FEVR] and Norrie disease [15-19]), genes with a known molecular genetic role in retinal vascularization or macular pigment transport, and genes of functional interest that lay in regions of interest based on familial linkage studies. Twenty-seven genes were identified and screened based on these criteria.

## Methods

### Study population

Participants were enrolled at 23 centers in seven countries (Australia, Germany, France, the UK, Israel, Switzerland, and the United States). Each center received approval from their governing human subjects review board. Informed consent was obtained in accordance with the Declaration of Helsinki. Records of informed consent and human subject approvals for all participating centers were centrally managed by the EMMES Corporation. Study subjects were enrolled based on a diagnosis of MacTel by the principal investigator at each center. Further criteria for enrollment were that patients be at least 18 years of age, be of European ancestry, and be free of diabetic retinopathy or other retinal disease [20]. Relatives of patients diagnosed with MacTel were actively recruited. Age- and ethnicity-matched controls were concurrently recruited—often a spouse or other unrelated individual present at the clinic appointment with the proband. At the time screening was performed, the MacTel cohort consisted of DNA from 200 unrelated probands diagnosed with MacTel. Since March 2010, study enrollment has been ongoing; a total of 735 samples have been sent to Columbia University’s Center for Human Genetics. Of those samples, 360 are unrelated cases, and the rest are mostly unaffected relatives. The youngest MacTel proband in the study was 25 years old at the time of enrollment, and the oldest was 85. The majority of probands were between 50 and 70 years of age when they were enrolled.

**Figure 1:**
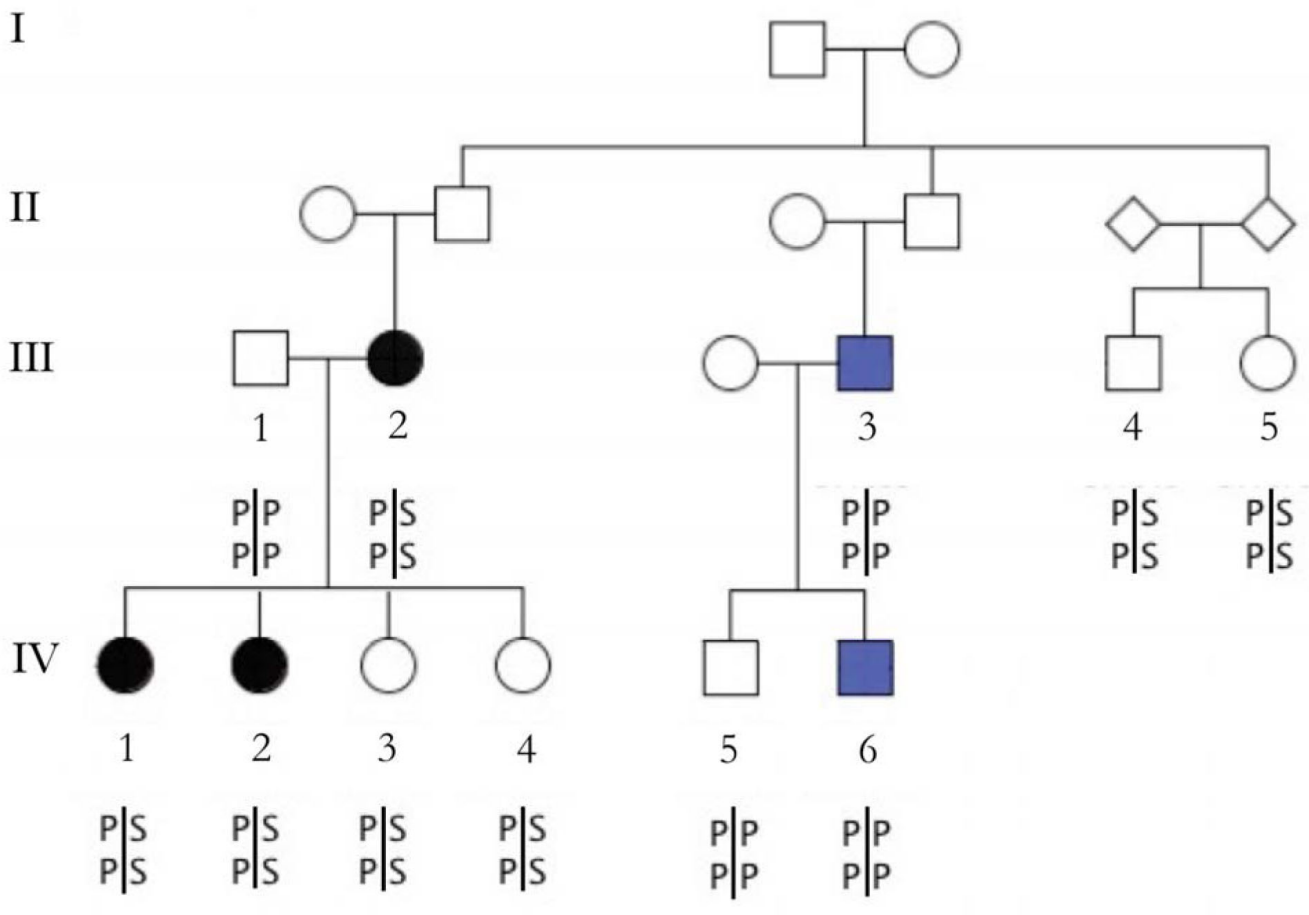
Segregation of the p.Pro33Ser (top)/p.Pro168Ser (bottom) compound variant in a family with inherited macular telangiectasia type 2. Black filled circles represent affected family members; blue filled circles represent possibly affected family members. The numbered individuals are those for whom DNA was available for analysis.

The families of the probands sequenced consisted of five affected sibling pairs (ASPs), two affected sibling trios, one affected parent and child duo, and additional relatives. Specifically, family 8 (Figure 1) consisted of 11 individuals, including an ASP, two additional unaffected siblings, one affected and one unaffected parent, two uncles, and three cousins. Family 9 consisted of an ASP and three unaffected and one possibly affected offspring of the siblings. Families 22, 30, and 81 were ASPs. Family 29 was an affected sibling trio plus six unaffected adult offspring of the siblings. Family 42 was a three-generation family consisting of an affected trio, an affected uncle, three unaffected individuals in the second generation, and three unaffected adult offspring in the third generation. Family 101 was a discordant sibling pair with one affected and one unaffected parent.

The Columbia University control cohort consisted of DNA samples from individuals recruited as controls for studies of age-related macular degeneration (AMD). Participants were matched by age and ethnicity to the AMD cohort, and were found free of macular disease at the time of recruitment, as previously described [21,22]. Briefly, controls underwent ophthalmic examination to screen for retinal disease. Stereo fundus photographs were evaluated using standard classification systems. Individuals accepted as controls had no family history of AMD, and were determined to be free of retinal disease. Three hundred and sixty-eight controls from the Columbia cohort were screened for this study. In addition, a cohort of 639 AMD patients was screened for selected variants. Data obtained from screening the AMD cohort was beneficial in that the patients in this cohort were of advanced age and had undergone thorough retinal examination, thereby ruling out MacTel and increasing the number of individuals classified as controls.

### Diagnosis of MacTel

Participants were given a standardized ophthalmic examination, including best corrected visual acuity, fundus examination with photography, fluorescein angiography, optical coherence tomography, and blue light reflectance. Images were taken and sent to Moorfields Eye Hospital’s Reading Centre, in London, England, for evaluation. The criteria for diagnosis are described by Clemons et al. [20]. Diagnostic features of MacTel are based on the Gass and Blodi criteria [1], and include loss of transparency in the perifoveal region, dilated and telangiectatic blood vessels, especially in the temporal retina, and crystalline deposits. In cases where the adjudication made at the reading center was not in accordance with the diagnosis made at the recruiting center, the reading center diagnosis was used to code the sample for genetic studies. Each sample was assigned to one of four diagnostic categories: affected, possibly affected, probably not affected, or unaffected. Patients were reevaluated at regular intervals over the course of the study.

### Sequencing and genotyping

DNA was isolated from whole blood by column purification (DNA Blood Maxi, 51194; Qiagen, Valencia, CA). Eight probands of families with more than one affected individual were screened by direct Sanger sequencing for mutations in 27 candidate genes. For each gene, primers were designed to amplify each exon and flanking intronic sequences. Primer sequences are listed in Appendix 1. PCR reactions were performed with 2 ng of genomic DNA in a total volume of 25 μg, using 25 pmol each of forward and reverse primer, 200 μM dATP, dCTP, dGTP, dTTP, 2.5 mM MgCl_2_, 1.5 U Taq Polymerase (Hot Fire DNA Polymerase, Solis Biodyne, Tartu, Estonia, or AmpliTaq Gold, Applied Biosystems, Carlsbad, CA), and 10× buffer supplied by the manufacturer. Thermocycling was performed using either the Stepdown protocol, or the Touchdown (68–55 °C) protocol. Stepdown: an initial 12 min denaturation step at 95 °C was followed by 12 cycles of 95 °C for 12 s, 65 °C for 20 s (with a 0.5 °C reduction in temperature for each cycle), and 72 °C for 55 s. This was followed by 30 cycles of 95 °C for 12 s, 50 °C for 20 s, and 72 °C for 55 s, with a final 7 min extension at 72 °C. Touchdown (68–55 °C): an initial 12 min denaturation step at 95 °C was followed by 26 cycles of 95 °C for 15 s, 68 °C for 20 s (with a 0.5 °C reduction in temperature for each cycle), and 72 °C for 45 s. This was followed by 15 cycles of 95 °C for 15 s, 55 °C for 20 s, and 72 °C for 45 s, with a final 7 min extension at 72 °C. Sequencing was performed by Genewiz (South Plainfield, NJ). One hundred and twelve familial samples were genotyped on the Illumina (Illumina, Inc., San Diego, CA) 1M chip for ongoing linkage studies (data not shown). These data were used to evaluate the segregation of variants detected by sequencing when the variant was a known single nucleotide polymorphism (SNP) that was genotyped on the chip.

Sequences were analyzed for known or unknown variants that differed from the reference sequence. Nonsynonymous coding variants were evaluated using the population frequencies published in the Single Nucleotide Polymorphism database (dbSNP). Unknown, infrequent, or disease-associated variants were evaluated to determine whether they co-segregated with the disease within the families in which they were discovered.

For known variants that were present in the Illumina 1M chipset, genotypes of relatives in the family were inspected to determine whether the variant co-segregated with the disease. For unknown variants, and for rare variants not included on the 1M chip, tests for co-segregation were performed by sequencing the family members of the proband in whom the variant was detected. For variants detected in a family with vertical transmission, where both parents were available, segregation was assessed based on whether the allele was inherited from the affected or the unaffected parent. For variants detected in affected sib pairs whose parents were not available, if the variant was present in both siblings, follow-up analysis was performed by TaqMan assay (Applied Biosystems, Foster City, CA) in the cohort of MacTel probands and in controls, to determine allele frequencies in these cohorts. Variants that were determined to be common polymorphisms based on population frequencies from dbSNP were not pursued further. In some cases, population frequencies were not available in dbSNP, yet the variant was highly polymorphic in the genotyped cohort and was present in both affected and unaffected individuals. Such variants were classified as frequent variants and not pursued further. Variants that merited further analysis either because co-segregation of the variant with the disease could not be ruled out, or because the allele detected was rare or had been associated with disease, were screened by TaqMan assay in the entire MacTel cohort, and in a cohort of controls. For selected variants, we also screened an available cohort of AMD patients to determine allele frequencies more precisely. Analyzing the large AMD cohort, in addition to the MacTel and control cohorts, provided additional allele frequency data for previously unknown variants and known variants where population frequency data was unavailable. Allele and genotype frequencies were compared between cases and controls with standard statistical tests, such as a 2x2 contingency table and Fisher’s exact text.

## Results

A summary of the screened genes grouped by selection criteria is shown in Table 1. Descriptions of the genes screened and the rationale for selecting candidate genes were as follows.

**Table 1:** Genes sequenced and missense variants detected by Sanger sequencing in 8 macular telangiectasia probands.

**Gene/ category**	**Variant**	**rs number**	**MAF MacTel cases (400 chromosomes) /MAF AMD cases (1278 chromosomes)**	**MAF Controls (736 chromosomes)**	**MAF dbSNP**	**Notes**
**Vascular / angiogenic**						
*AGGF1*	p.Pro698Thr	rs34400049	NS	NS	0.28	FV, DNS
*ANG1*	p.Thr257Arg	-	0.005 (1/400)	0 (0/736)	-	DNS
*DKK1*	None					
*FZD4*	p.Pro33Ser	rs61735304	0.02 (6/400)/.03 (17/1278)	0.01 (13/736)	ND	DNS, MT, AMD
	p.Pro168Ser	-	NS	NS	-	Allelic with P33S
*HIF1A*	None					
*LRP5*	p.Val667Met	rs4988321	0.07 (28/400)	0.06 (41/736)	0.03	MT, DNS
	p.Gln1192Arg	-	0.005 (1/400)	0 (0/736)	-	MT, DNS
	p.Ala1130Val	rs3736228	NS	NS	0.12	FV, DNS
*NDP*	None					
*PEDF*	p.Thr72Met	rs1136287	0.37 (148/400)/.34 433/1278)	0.33 (245/736)	0.36	FV, MT, AMD
*THBS1*	p.Thr523Ala	rs2292305	NS	NS	0.1	FV, DNS
*TIE2*	p.Val486Ile	rs1334811	NS	NS	0.03	DNS
	p.Val600Leu	rs35030851	NS	NS	0.04	DNS
*VHL*	None					
**Expression microarray**						
*AGTRL1*	None					
*APLN*	None					
*CFB*	p.Leu9His	rs4151667	0.04 (16/400)/.02 (23/1278)	0.04 (29/736)	0.07	DNS, MT, AMD
	p.Arg32Gln	rs641153	0.10 (40/400)/.04 (51/1278)	0.12 (88/736)	0.1	DNS, MT, AMD
	p.Gly252Ser	rs4151651	NS	NS	0.04	DNS
	p.Lys533Arg	-	0.04/.02 (23/1278)	0.04 (29/736)	-	DNS, AMD, LD
*LRG1*	p.Pro133Ser	rs966384	NS	NS	0.35	DNS, FV
*PLVAP*						
**Macular pigment**						
*GSTP1*	p.Ile105Val	rs1695	0.33 (132/400)/.30 (383/1278)	0.3	0.39	MT, AMD, FV
	p.Ala114Val	rs1138272	0.08 (32/400)/.07 (87/1278)	0.06 (46/736)	0.12	MT, AMD, FV
*SCARB1*	p.Val135Ile	rs5891	0.03 (12/400)	0.02 (15/736)	0.01	MT DNS
	p.Pro376Leu	rs74830677	0.01 (3/400)	0.01 (5/736)	0.05	MT, DNS
**Genes under suggestive linkage peaks**						
*CCM2*	p.Val120Ile	rs11552377	NS	NS	0.17	FV, DNS
*IGFBP3*	None					
*SSPN*	None					
*TGFB2*	None					
**Disease related genes**						
*AGTR1*	p.Ala244Ser	rs12721225	NS	NS	0.03	DNS
*ALDH3A2*	None					
*OXGR1*	p.Thr205Ala	-	0.005 (1/400)	0 (0/736)	-	DNS
*SUCNR1*	None					
*VLDLR*	None					

NS represents not screened, ND represents not determined, FV represents frequent variant, DNS represents Does not segregate with disease, MT represents Screened in MacTel cases and controls, AMD represents Screened in AMD cohort. LD represents the *CFB* p.Lys533Arg variant is in complete linkage disequilibrium with p.Leu9His. No comparison between MacTel cases and controls reached statistical significance.

### Genes involved in angiogenesis

One of the defining phenotypes of MacTel is the presence of telangiectatic blood vessels in the retina and, in advanced disease, intraretinal neovascularization. This form of neovascularization is prevalent in diabetic retinopathy and in retinopathy of prematurity (ROP), and represents about 10%–15% of neovascular changes in AMD [23-27]; the remaining 85%–90% of neovascularization in AMD involves aberrant vessels arising from the choroidal vasculature (CNV). The vasculature of the retina is highly specialized; thus, the specific location of the aberration is likely a result of highly tissue-specific molecular genetic interactions. We selected 11 genes related to angiogenesis (Table 1), including the Wnt receptor frizzled-4, its ligand, norrin (*NDP*), and the co-receptor, low-density lipoprotein receptor-related protein 5 (*LRP5*). Mutations in these genes have been implicated in FEVR, Norrie disease, and ROP [28-30]. Mouse knockout models of frizzled homolog 4 (*Drosophila*); (*FZD4*) [15] and NDP [31] show a lack of intraretinal vessel formation. Other genes involved in angiogenesis or vessel regulation that were screened are angiogenic factor with G patch and FHA domains 1 (*AGGF1*) [32], angiopoietin 1 (*ANG1*) [33], dickkopf homolog 1 (*Xenopus laevis*); (*DKK1*) [34], hypoxia inducible factor 1, alpha subunit (basic helix–loop–helix transcription factor); (*HIF1A*) [35], serpin peptidase inhibitor, clade F (alpha-2 antiplasmin, pigment epithelium derived factor), member 1 (*PEDF*) [36], thrombospondin 1 (*THBS1*) [37], tyrosine kinase, endothelial (*TIE2*) [38], and von Hippel-Lindau tumor suppressor (*VHL*) [39].

### Genes involved in macular pigment transport

MacTel is also characterized by macular pigmentary changes, with advanced cases often lacking macular pigment altogether. Little is known about the molecular genetics of macular pigment transport in the retina. In the healthy retina, the two macular pigments, lutein and zeaxanthin, filter damaging blue light [40,41]. The proteins responsible for lutein transport are unknown. The macular pigment genes screened were glutathione S-transferase pi 1 (*GSTP1*), a binding protein for zeaxanthin [42], and scavenger receptor class B, member 1 (*SCARB1*), which has been proposed as a lipid transporter in the retina [43].

### Genes identified from expression arrays

Several genes were screened that were identified as differentially expressed in mouse models intended to mimic some aspects of the MacTel phenotype (data not shown). From the top of this list, five genes were screened that were identified as also having possible functional relevance by playing a role in MacTel: apelin receptor (*AGTRL1*), apelin (*APLN*) [44], complement factor B (*CFB*) [45], leucine-rich alpha-2-glycoprotein 1 (*LRG1*), and plasmalemma vesicle associated protein (*PLVAP*) [46].

### Genes identified from suggestive linkage regions

Linkage studies were performed using families in which at least one family member in addition to the proband was diagnosed as affected by MacTel. While these results will be reported separately, several genes of possible functional interest were identified in regions of suggestive linkage on chromosomes 1, 7, 10, and 12 during the course of analysis. Cerebral cavernous malformation 2 (*CCM2*) [47], insulin-like growth factor binding protein 3 (*IGFBP3*) [48], sarcospan (Kras oncogene-associated gene); (*SSPN*) [49], and transforming growth factor, beta 2 (*TGFB2*) [50] were selected and screened as candidates under these criteria. Each of these genes has been proposed to be involved in angiogenesis or regulation of blood vessels; *IGFBP3* and *TGFB2* have also been proposed as genes related to diabetes. Linkage studies are ongoing as additional families are recruited.

### Genes involved in diseases with related phenotypes

An increased prevalence of hypertension and diabetes are found in MacTel patients [51]. Genes involved in these diseases, which are also expressed in the retina, especially in the vasculature, were considered as candidates. Succinate receptor 1 (*SUCNR1*) [52], angiotensin II receptor, type 1 (*AGTR1*) [53], aldehyde dehydrogenase 3 family, member A2 (*ALDH3A2*) [54,55], very low density lipoprotein receptor (*VLDLR*) [56], and oxoglutarate (alpha-ketoglutarate) receptor 1 (*OXGR1*) were screened on this basis, in conjunction with linkage or expression array data, or personal communication with collaborators.

Table 1 summarizes the variants detected by the complete sequencing of all exons and adjacent intronic sequences in 27 candidate genes in eight MacTel probands. In total, we discovered three unknown and 20 known missense changes, and 22 synonymous and 61 intronic variants. Frequent variants with reported minor allele frequencies (MAFs) over 0.10 were not analyzed further unless the variant was reported to be disease-associated (*PEDF* p.Thr72Met and *GSTP1* p.Ile105Val were screened in cases and controls, though they are frequent variants). Variants with published population frequencies between 5%–10% were assessed for further screening, based on whether the variant had been reported to be associated with any diseases with phenotypes similar to that of MacTel. Twelve missense variants were screened by TaqMan assay (Table 1) in MacTel cases and unaffected controls; six of these variants were also screened in a large AMD cohort. Of the variants detected, three were unknown (*ANG1* p.Thr257Arg, *LRP5* p.Gln1192Arg, and *SCARB1* p.Ile135Val), three had been reported as possibly disease-associated (*FZD4* p.Pro33Ser, *GSTP1* p.Val105Ile, and *PEDF* p.Thr73Met). The remainder had low reported MAFs. None of the variants found by sequencing segregated with the disease. Of the variants screened by TaqMan assay, only *GSTP1* p.Val105Ile showed a trend toward a statistically significant frequency difference between cases and controls (p=0.09), suggesting it could be a possible modifier, but not a causal gene for MacTel.

The *FZD4* variants p.Pro33Ser and p.Pro168Ser were detected in the proband III2 (family 8, Figure 1). We sequenced all members of family 8 and found both the p.Pro33Ser and p.Pro168Ser variants present in two affected daughters, one unaffected daughter, and one unaffected cousin of the proband, indicating that the complex allele containing both mutations did not segregate with the disease (Figure 1). The p.Pro33Ser variant was analyzed by TaqMan assay in 200 MacTel cases, 368 unrelated controls, and 639 AMD cases to determine allele frequencies. This variant was found in one other unrelated MacTel proband (A5). The p.Pro168Ser mutation was also present in each individual carrying p.Pro33Ser. Thirteen controls were heterozygous for p.Pro33Ser (MAF=0.018). In 639 unrelated AMD samples, 16 heterozygotes and one homozygote for p.Pro33Ser were detected (MAF=0.013). In conclusion, there was no statistically significant difference in allele frequencies between cases and controls.

## Discussion

We have shown that the *FZD4* p.Pro33Ser /p.Pro168Ser complex allele, which has been reported as causative in FEVR and ROP [57,58], is present in 2% of unaffected controls, and therefore is not a disease-causing variant in MacTel, FEVR, or ROP. The same allele was detected in one MacTel patient, prompting the hypothesis that FEVR and MacTel may be allelic diseases. Given that FEVR has phenotypic similarities to MacTel, in that the intraretinal vascular plexus is perturbed in both diseases, dysregulation of *FZD4* was a plausible hypothesis in the etiology of MacTel. Both FEVR and MacTel also exhibit variable expressivity [59]. In both diseases, affected family members are often unaware that they are affected until a diagnosis is made after thorough examination. Segregation analysis in one MacTel family and case-control association analysis using a large cohort of controls revealed that p.Pro33Ser /p.Pro168Ser, which had been reported as a disease causing mutation, rather, is a benign polymorphism present at a low frequency in the general population. Accordingly, we conclude that it is not causative in either MacTel or FEVR, because our control cohort had no documented retinal disease. This result highlights the importance of segregation analysis in families, and of screening a sufficient number of controls to distinguish between causal mutations and rare benign polymorphisms.

Candidate gene screening is a widely used method for detecting disease-associated variants and genes. While often criticized as a “needle in a haystack” approach, it has been successful in determining some disease-associated genes, most notably the major AMD-associated genes, *CFH* and *CFB* [21,22]. In this study, 27 possible candidate genes for MacTel were selected based on a combined set of criteria. The exons and flanking intronic regions of all genes were screened by direct sequencing, with follow-up segregation analysis in families and TaqMan genotyping in large cohorts for rare, unknown, or previously disease-associated missense variants. No variants segregated with the disease, and none showed significant association with MacTel, allowing exclusion of the coding regions of these genes as harboring a causal mutation for MacTel. The causal gene(s) for MacTel are currently being searched for by a combination of linkage analyses and whole-genome sequencing.

## Appendix 1. Primer sequences of candidate genes sequenced (5′ −3′).

To access the table, click or select the words “Appendix 1.” This will initiate the download of a pdf archive that contains the table.
